# Abundance and distribution of microplastics within surface sediments of a key shellfish growing region of Canada

**DOI:** 10.1371/journal.pone.0196005

**Published:** 2018-05-23

**Authors:** T. N. Kazmiruk, V. D. Kazmiruk, L. I. Bendell

**Affiliations:** Ecotoxicology Research Group (ERG), Department of Biological Sciences, Simon Fraser University, Burnaby, British Columbia, Canada; Centro de Investigacion Cientifica y de Educacion Superior de Ensenada Division de Fisica Aplicada, MEXICO

## Abstract

The abundance and distribution of microplastics within 5 sediment size classes (>5000 μm, 1000–5000 μm, 250–1000 μm, 250–0.63 μm and < 0.63 μm) were determined for 16 sites within Lambert Channel and Baynes Sound, British Columbia, Canada. This region is Canada’s premier growing area for the Pacific oyster (*Crassostrea gigas*). Microplastics were found at all sampling locations indicating widespread contamination of this region with these particles. Three types of microplastics were recovered: microbeads, which occurred in the greatest number (up to 25000/kg dry sediment) and microfibers and microfragments, which were much less in number compared with microbeads and occurred in similar amounts (100–300/kg dry sediment). Microbeads were recovered primarily in the < 0.63 μm and 250–0.63 μm sediment size class, whereas microfragments and microfibers were generally identified in all 5 sediment size classes. Abundance and distribution of the three types of microplastics were spatially dependent with principal component analysis (PCA) indicating that 84 percent of the variation in abundance and distribution was due to the presence of high numbers of microbeads at three locations within the study region. At these sites, microbeads expressed as a percent component of the sediment by weight was similar to key geochemical components that govern trace metal behavior and availability to benthic organisms. Microbeads have been shown to accumulate metals from the aquatic environment, hence in addition to the traditional geochemical components such as silt and organic matter, microplastics also need to be considered as a sediment component that can influence trace metal geochemistry. Our findings have shown that BC’s premier oyster growing region is highly contaminated with microplastics, notably microbeads. It would be prudent to assess the degree to which oysters from this region are ingesting microplastics. If so, it would have direct implications for Canada’s oyster farming industry with respect to the health of the oyster and the quality of product that is being farmed and sets an example for other shellfish growing regions of the world.

## Introduction

Microplastics have been defined as plastic particles ≤5 mm in length [1, 2, 3, 4] and depending on their origin, can be divided into two groups: primary, virgin granules originally constructed to be of microscopic size and used to produce macroplastics and secondary, originating from the degradation of macroplastics [5]. Global production of plastic has increased from approximately 5 million tons per year during the 1950s to over 280 million tons in 2016 and is expected to grow at a rate of 9 percent per year, as India, China, and the African continent begin to discover the benefits and advantages that plastics offer [6,7]. Not surprisingly, plastics are reported to be the main contributor to marine and beach litter (between 60 and 80 percent) [5, 8, 9, 10, 11] and occur in concentrations of 3 particles per m^3^ in water [12] and 15 particles per kg in surface sediments [13], to hot-spot concentrations of 9200 particles m^3^ in water [13] and 621,000 particles per kg in sediments [14]. Nine types of polymers have been identified in intertidal sediments: acrylic, alkyd, polyethylene, polypropylene, polyamide (nylon), polyester, polymethyl acrylate, and polyvinyl-alcohol [4, 15]. These have a wide range of uses, including clothing, packaging, rope, basic household items, personal care products, agriculture and industry.

Aquatic sediments have long been the repository of metals introduced into aquatic environments. Understanding the role of sediment geochemistry in influencing metal behavior has been an extensive area of study since the early 1970s because sediments, in addition to acting as a sink, can also be a source of metals to sediment-dwelling organisms. The sediment can therefore provide an entry of potentially toxic metals to the base of food webs. Zhang et al. [16] recently reviewed the literature on the effects of sediment geochemical properties on heavy metal bioavailability and noted that under anoxic conditions, acid-volatile sulfides reduce solubility and hence toxicity of metals, whereas oxides and iron and manganese, organic matter, clay and silt can stabilize metals in oxic environments.

Important was the recognition that trace metal availability to sediment ingesting organisms was to some extent dependent on sediment geochemistry. Microplastics present in sediments relative to other key sediment components that govern metal behavior could also influence metal bioavailability. For example, microplastics within sediments from the Rhine River can account for 0.1 percent by weight of the sediment [17] which is an amount similar to percent organic matter and silt, two key sediment components that govern trace metal availability of benthic invertebrates.

Although much has been documented on the abundance, fate and distribution of microplastics within marine and freshwater sedimentary environments [18,19] still not well known is the abundance and distribution of different types of microplastics within sedimentary environments relative to each other. As each type of microplastic will have its own unique physical properties, such information would aid in linking processes that influence the distribution and depositional behavior of microplastics within sediments.

The objectives of the current study are two fold: 1) to assess the abundance in terms of both number and mass, and the distribution of different microplastic types within a defined geographical area and 2) to determine the relative importance of microplastics as a sediment component compared with the traditional sediment descriptors and predictors of sediment metal bioavailability, percent by weight of silt and organic matter. To meet our objectives we sampled sediments for microplastics, grain size and organic matter within Baynes Sound and Lambert Channel, British Columbia (BC), Canada’s premier shellfish growing region. The sound is a geographically defined region where hydrodynamic processes that govern microplastic behavior could possibly be identified. Recovered amounts of microplastics expressed as a percent by weight of sediment could also be compared with percent organic matter and silt to assess the significance of their contribution to the sediment matrix. Finally, shellfish such as oysters and mussels have been shown to ingest microplastics, both beads and fibers [20] which have been shown to be a source of pollutants to shellfish [21], as well as negatively affect shellfish reproductive output [22]. An important outcome of our findings will be to assess the risk that microplastics pose to shellfish within a region used exclusively for shellfish aquaculture.

## Materials and methods

### Study area

**Fig 1:**
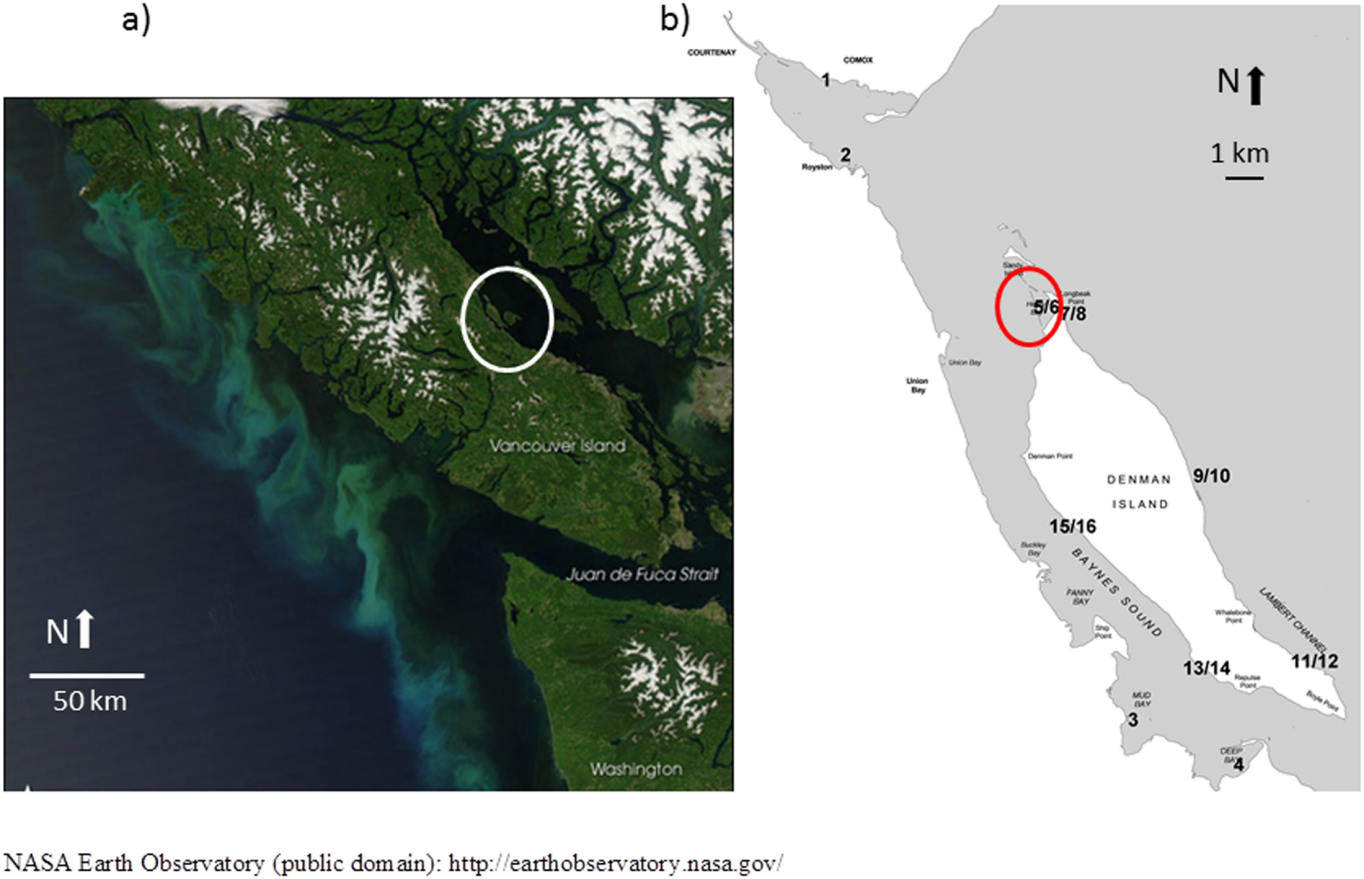
(a and b.) Location of the study site, Baynes Sound and Lambert Channel (Lat: 49° 31’59.99” N Long: -124 ° 49’ 59.99” W) (a), and study site sampling locations (b). Sample sites are as follows: 1 Comox Estuary Low Tide, 2 Royston Bay Low Tide, 3 Mud Bay Low Tide, 4 Deep Bay Low Tide, 5/6 Henry Bay Low and High Tide, 7/8 Morningside Beach Low and High Tide, 9/10 Fillongley Park Low and High Tide, 11/12 Gravelly Bay Low and High Tide, 13/14 Metcalfe Bay Low and High Tide, Ferry Terminal Low and High Tide 15/16. Sites 1–6 and 13–16 are within Baynes Sound, whereas sites 7–12 are within Lambert Channel. The shallow underwater plateau is indicated with a red circle [24].

Baynes Sound and Lambert Channel fall within the Regional District of Comox Strathcona and include the foreshore of the City of Courtenay and the Town of Comox [23]. The sound is ca. 25 km long and 3.5 km wide at its widest point, with the average width being 2 km. Baynes Sound has over 9000 ha of shallow coastal channel fringed by protected bays, open foreshore, tidal estuaries, and inshore marshes. Comox Harbour is one of the largest low gradient deltaic deposits on the east coast of Vancouver Island (Fig 1a). An important feature of the sound is the Comox Bar, a shallow underwater plateau that extends north of Denman Island which reduces circulation at the north end of Baynes Sound, with this portion of the sound behaving like an embayed water body rather than an open channel (Fig 1b) [24]. Tides are mixed and mainly semi-diurnal. Tidal range at Comox BC ranges from 0.1m to 5.4 m above chart datum [25]. Baynes Sound has a long history of shellfish aquaculture dating back to the 1900’s [23] with the Pacific oyster *Crassostrea gigas* 80 percent of the product farmed. In 2001, over 55 percent of the viable intertidal zone was used exclusively for shellfish farming purposes [23].

Field sampling was conducted along the east coast of Vancouver Island (Deep Bay, Mud Bay, Buckley Bay, Royston Estuary, and Comox Estuary), west coast of Denman Island (Henry Bay, Ferry Terminal and Metcalfe Bay), and Lambert Channel on the east coast of Denman Island (Morningside Beach Park, Fillongley Provincial Park and Gravelly Bay). Sample sites were as follows: 1 Comox Estuary Low Tide (CVLT), 2 Royston Bay Low Tide (RLT), 3 Mud Bay Low Tide (MBLT), 4 Deep Bay Low Tide (DBLT), 5/6 Henry Bay Low and High Tide (HBLT, HBHT), 7/8 Morningside Beach Low and High Tide (MSLT, MSHT), 9/10 Fillongley Park Low and High Tide (FPLT, FPHT), 11/12 Gravelly Bay Low and High Tide (GBLT, GBHT), 13/14 Metcalfe Bay Low and High Tide (MBLT, MBHT), Ferry Terminal Low and High Tide 15/16 (FTLT, FTHT) (Fig 1b). Sites 1–6, 13–14 are in Baynes Sound, whereas sites 7–12 are located within Lambert Channel (Fig 1b). Field sampling of sediments occurred on Provincial and Crown Land. Permits are required for the sampling of invertebrates and fish, none are required for sediment collection. No endangered species were compromised by sample collection.

### Sediment sampling

Sampling was conducted during the period of maximum low tides that occur in late June. At each site two sets of sub- samples were collected, one for sediment characteristics of grain size and organic matter content and one for microplastic analyses. Intertidal surface sediment were collected from the top oxic 5 cm of sediment in the intertidal zone at the low and high tide mark at each site except for sites 1, 2 and 3 where only low tide samples were collected. Replicate sediments samples were taken from 3 to 5 separate 0.5 m x 0.5 m quadrats, located 3–5 m apart in undisturbed areas using a clean stainless steel spatula [26,27]. All replicate sediment samples at each point were pooled to form one composite sample (a total of 32 composite sediment samples, 16 for microplastics, 16 for sediment characteristics), were packed into pre-labelled sealed freezer bags, transferred to the laboratory within 48 hrs. and stored at—20 °C until required for analysis.

### Sediment analyses

Sediments were defrosted and air dried at room temperature, gently homogenized by mortar and pestle and then stored at 4°C prior to analysis.

### Grain size

Sediments were separated into 5 different size factions; >5000 μm (coarse gravel), 5000–1000 μm (gravel), 1000–250 μm (sand), 250–0.63 μm (fine sand), and < 0.63 μm (fine silts and clays) after methods of Mudroch et al. [28]. The different size fractions were dried and converted to percent sediment composition on a dry weight basis. After each sample was processed, sieves were rinsed and cleaned to ensure no cross contamination from one sample to the next.

### Organic matter

Percent sediment organic matter was determined by "loss-on-ignition" (LOI) [28]. Dried sediment subsamples of 1.0 g– 2.0 g were ignited at 400°C–440°C (to avoid the destruction of any inorganic carbonates in the sediments) for 5–10 hrs.

### Microplastics

Sediments were dried at air temperature, gently homogenized then sieved into 5 size fractions (>5000 μm, 5000–1000 μm, 1000–250 μm, 250–0.63 μm, and <0.63 μm). Microplastic particles were extracted from sediment using the flotation method described by Thompson et al. [4] with some modifications. Saturated saline (NaCl and sea salt) solutions with a density of 1.35 g/cm^3^ (360 g NaCl/l H_2_O- solubility of NaCl at 25°C) was used to separate microplastics from sediment. To determine the optimum amount of subsample for the extraction of microplastic particles from the sediment fractions, experiments with different weights of subsample of different grain sizes were performed. From these preliminary experiments subsamples of 100.0 g (grain size >5000 μm), 75.0–50.0 g (grain size 1000–250 μm), 50.0–30.0 g (grain size 250–0.63 μm), and 15.0–10.0 g (grain size of <0.63 μm) were determined as appropriate. Microplastics captured within the largest grain size > 5000 μm are not microplastics as they are larger than 5 mm. However we wanted to include this size fraction to determine the amount of larger plastic debris that would be recovered from the surface sediments through the saline extraction procedure. Sieved sediment was transferred directly to Erlenmeyer^®^ flasks used for density separation and 250–900 ml (depending on volume of flask) of NaCl /sea salt solution were added. Sediment were stirred at high intensity for 20–25 min then allowed to settle for 12 to 24 hrs. depending on the observed clearance rate of the sediment from suspension. Prior to filtering, glass fiber filters (Whatman GF/A 47 mm diameter, GE Healthcare Whatman) were weighed (accuracy 0.0001 g) and rinsed with distilled deionized water (dd H_2_O) to minimize contamination. After the sediments settled, the supernatant was extracted by moving a 30–50 ml pipette across the solution surface, and expelling the pipette onto a glass fiber filter. The pipette was rinsed with distilled deionised water to capture all remaining small particles possibly adhering to the pipette walls. The remaining 250–500 ml supernatant was decanted and filtered through a separate glass microfiber filter by vacuum filtration. After the extracting of supernatant, an additional volume (250–500 ml) of NaCl solution was added to each sample, and after allowing sediments to settle the same procedure of filtration was repeated to extract any remaining microplastic particles. This extraction procedure was carried out 2–5 times for each sediments subsample. This procedure of filtration was applied to all 5 size fractions. After filtration, filters were dried at 50°C for 24 h. All filters were inspected under dissecting microscope at 40X magnification (Olympus SZ51) and all polymer material investigated by visual inspection was considered as potential microplastic particles or fibers. In addition the the hot needle test [29] was used as an alternative means to identify microplastic particles. Following visual inspection microplastic particles and fibers were analyzed under a dissecting stereo microscope equipped with the DP21 digital camera with a 10 fold magnification to facilitate the distinction between microplastic particles and natural sediment or organic matter, as well as between synthetic/anthropogenic and organic fibers. Potential microplastic particles, fragments and synthetic fibers were photo-catalogued with high magnification. An image analysis technique initially developed for the particle size analysis in sediments was used to count large numbers of microplastic beads, fragments, and fibers. All material visually identified as plastic was measured, counted and categorized into three different types: bead, fiber, and fragment [17]. Precautions were taken to minimize sample contamination, notably for microfibers (e.g., cotton lab coats worn throughout the procedure, clean and isolated work space). However, numbers of particles recovered from the sediment samples indicated that the primary particulates present were microbeads, which do not have the same degree of contamination concerns (e.g., false positives) as with microfibers.

#### Mass of microbead in surface sediments

The number of recovered microbeads was converted to a percent mass of sediment (for comparison with percent silt and organic matter by weight) as:

Mt=Vt*density,

where Mt is the total mass

Vt=V*N

where V = 4/3πr^3^ (volume of a sphere assuming a radius of 150 μm [30],

N is the number of recovered microbeads as #/kg, and density is either 0.9 g/cm^3^ or 1.34 g/cm^3^ i.e., minimum polymer density [reviewed in 24] and maximum polymer density based on the 1.35 density solution used to separate polymers in this study. To express values as a percent of sediment to allow for the comparison of % microbeads to % silt and % organic matter by weight, Mt is divided by 10 (g/kg/10 = g/100g or % microbead by weight).

### Statistical analysis

Recovered microplastics were expressed on a number basis (items of particles per one kilogram of dry sediment (items/kg) and items of particles per one square meter (with 1 cm of depth) (items/m^2^). Software used to analyze data included SigmaPlot 12 (Systat Software Inc.), and PRIMER v6 [31].

### Sediment analysis

Data were square root transformed (to reduce right skewness and to stabilize variance) [31]. As there were strong inter-correlations among the four grain size parameters, a principal component analysis (PCA) which takes into account data co-linearity was applied to sediment characteristics (percent grain size and organic matter) to determine which combination of sediment characteristics best accounted for most variability among the sampling sites (e.g., [32])

### Microplastic analysis

A multivariate analysis most commonly applied in ecological studies that address factors that affect the abundance and distribution of organisms was applied, PRIMER (Plymouth Routines in Multivariate Ecological Research) [31]. PRIMER provides a number of routines for analyzing data from community ecology but also can be applied to physical values and chemical concentrations [31]. In this application, we have applied a PCA and Bray Curtis similarity profiles to seventeen variables: the number of microbeads, microfibers and microfragments recovered in each of the 5 size classes, plus the total number of microplastics (the sum of all fractions). (No microbeads were recovered in the > 5000 μm mesh size, hence n = 17). In this application we are using routines available through PRIMER to determine, based on these 17 variables, which sites were most similar to each other. The ecological analogy would be “which site is most similar based on species composition”. Data was square root transformed (for reasons given above for the sediment characteristics) and a PCA applied to determine which combination of variables best accounted for the variability among the sampling sites. Bray Curtis similarity profiles were applied to show those sites which were most similar at 70 percent similarity.

## Results and discussion

While we know much about the abundance and distribution of microplastics as defined by those plastics < 5 mm in size [e.g. 17, 18], less is known about how different microplastics behave within marine sedimentary environments, specifically the abundance and distribution of different types of microplastics. Also unknown is how important microplastics are as compared to traditional sediment components such as percent weight of organic matter and silt, key determinants for the fate of metals within sedimentary environments. Here we applied a multivariate approach to determine the abundance and distribution of microplastics within Baynes Sound/Lambert Channel as well as assessing the importance of microplastics relative to percent silt and organic matter as a component of intertidal sediments. We use Baynes Sound/Lambert Channel as an example ecosystem, with our findings readily applicable to other sensitive ecosystems world wide.

### Sediment characteristics

**Fig 2:**
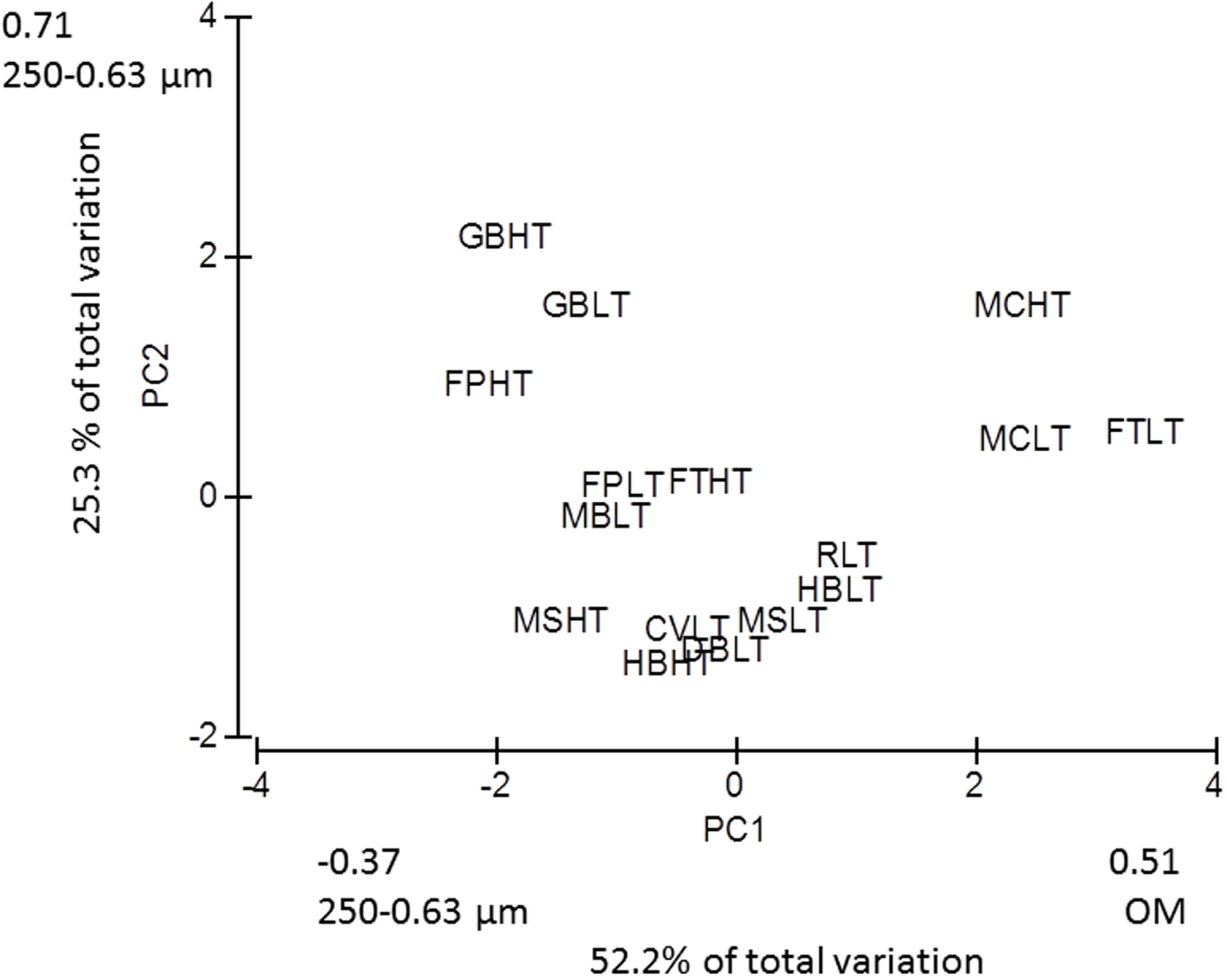
Principal component analysis for sediment characteristics of the 16 sampling sites. PC1 Principal component 1, PC2, Principal component 2, OM; organic matter. Location identifiers for sites within the bivariate plot are provided within the text within the Methods section.

Principal Component Analysis indicated that the 16 sampling locations grouped into 3 similar sediment types based on organic matter and 250–0.63 μm grain size. Metcalfe Bay and the Ferry Terminal Low and High Tide (sites 13/14 and 15/16) were characterized by high organic matter as compared to all other sites (Fig 2). Gravelly Bay, (sites 11/12) both Low and High Tide and Fillongley Park High Tide (site 9) were characterized by a grain size of 250–0.63 μm (Fig 2). Complete results of the PCA are presented in S1 Table.

### Microplastics: Characterization, distribution and abundance (number and mass)

**Fig 3:**
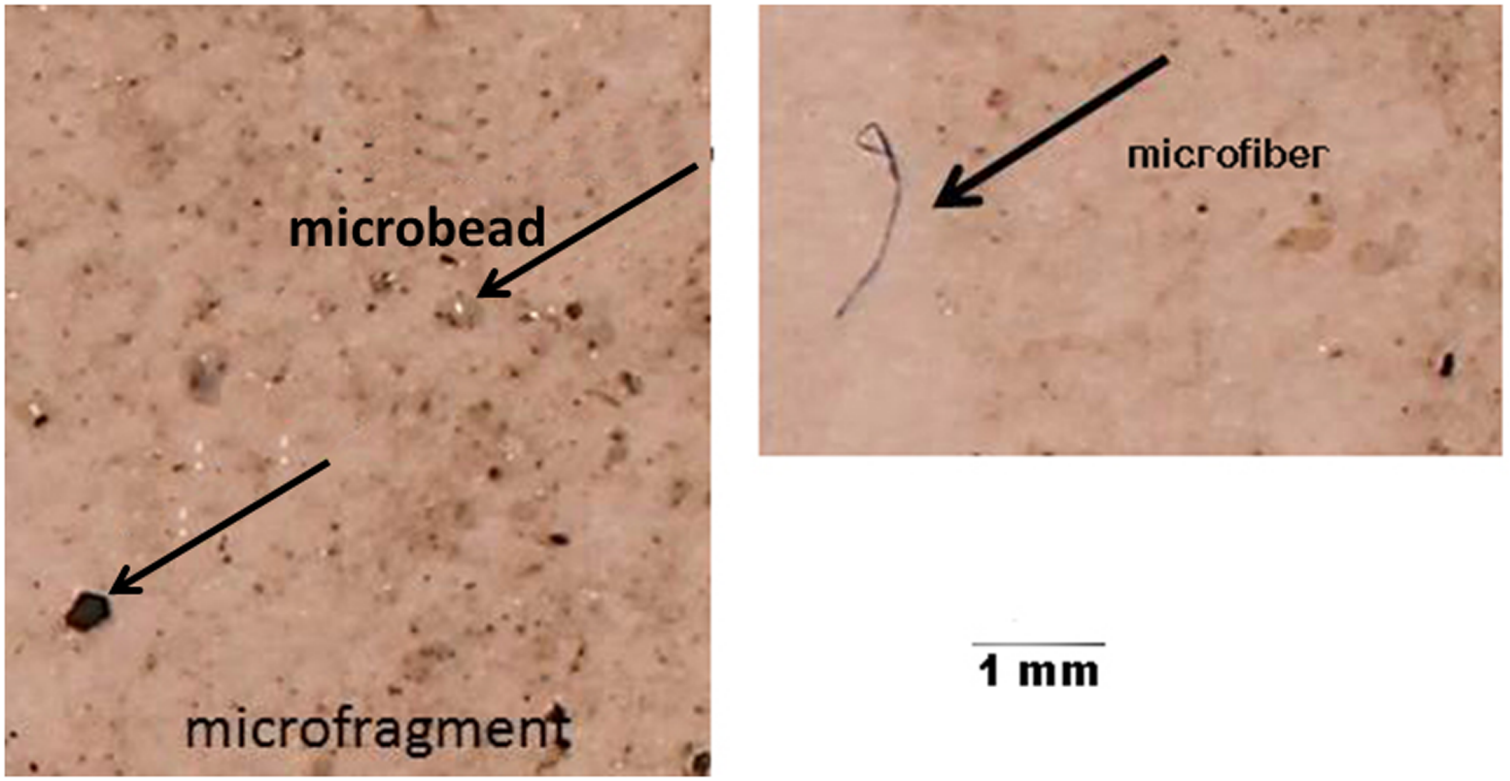
Examples of the types of microplastics recovered from sediments.

**Fig 4:**
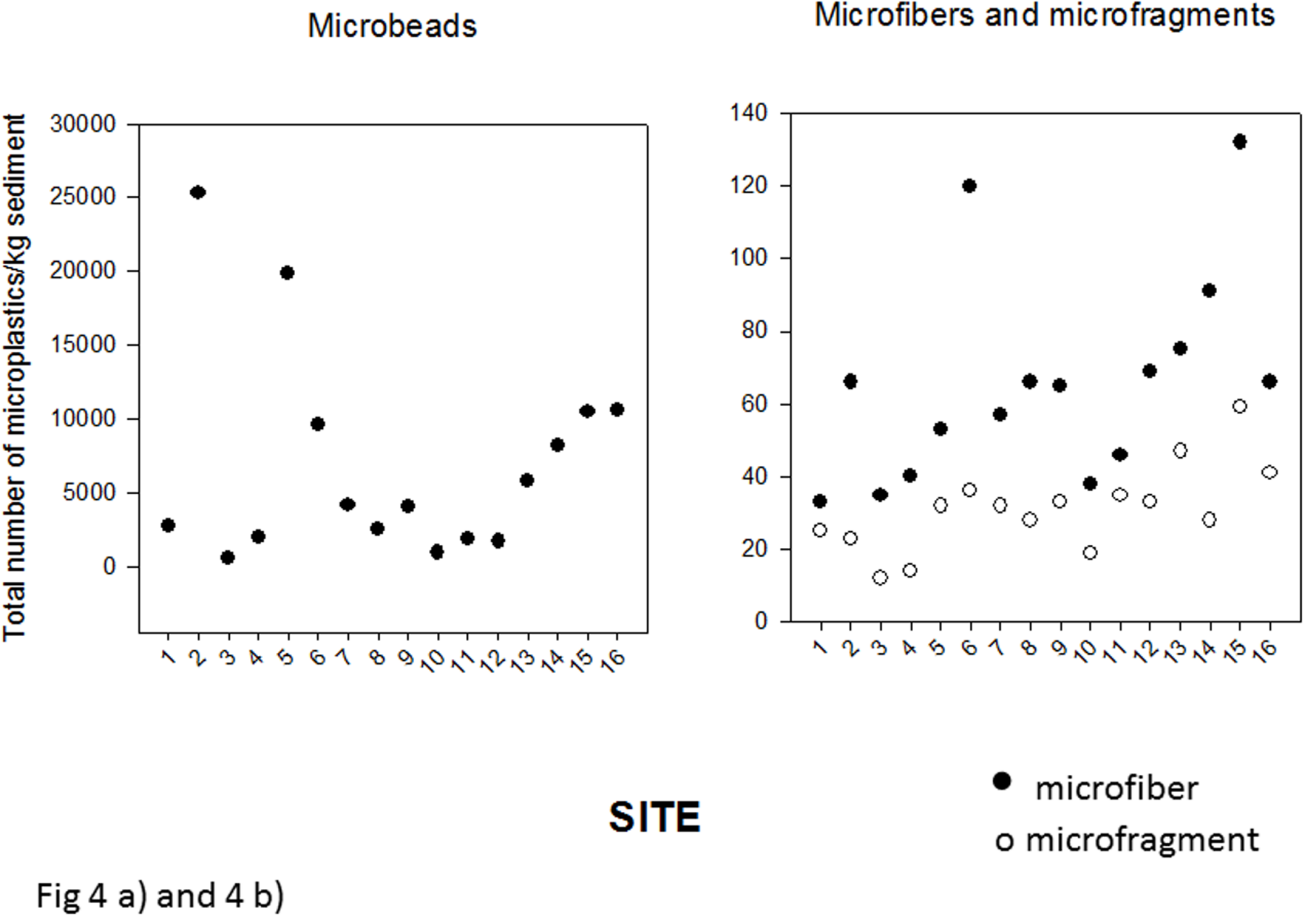
(a and b) Number of microbeads (a) and microfiber and microfragments (b) recovered from the intertidal sediments from the 16 sampling locations within Baynes Sound and Lambert Channel. Site name identifiers are provided within the text within the methods section.

Microplastics were found at all sampling locations within Baynes Sound (sites 1–6 and 13–16) and Lambert Channel (sites 7–12) indicating widespread pollution with these contaminants. Three main types of microplastics were identified: microfibers, microbeads and microfragments (Fig 3). The greatest numbers of all three types were found within Baynes Sound coincident with regions of intense shellfish aquaculture (Henry Bay, site 5 and Metcalfe Bay, site 13). Of the three types of microplastics, by far, microbeads were the most abundant with a maximum of 25,000/kg sediment recovered from Royston Low Tide (site 2) followed by Henry Bay (site 5) (Fig 4a and 4b). Greater numbers of microfibers were recovered from Henry Bay, Low Tide (site 5) and Ferry Terminal Low Tide (site 15) (Fig 4b) and sites 13 and 15 (Metcalfe Bay and Ferry Terminal Low Tide) contained the greatest number of microfragments (Fig 4a and 4b).

**Fig 5:**
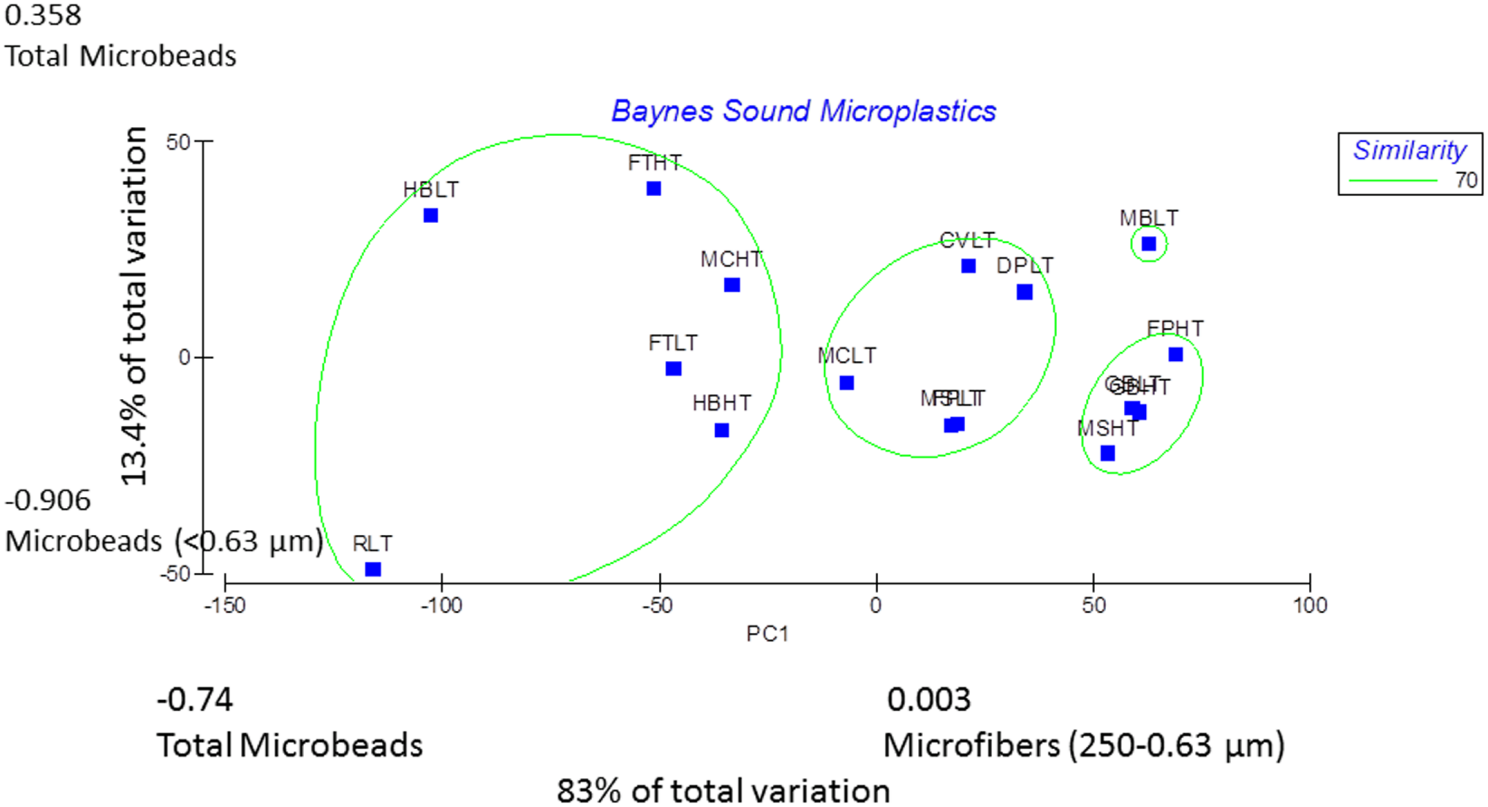
Principal component analysis for the microplastic characteristics of the 16 sampling sites. Those samples most similar at 70% are indicated by the green circles. The presence of microbeads accounts for the greatest amount of variation among all sample sites.

**Fig 6:**
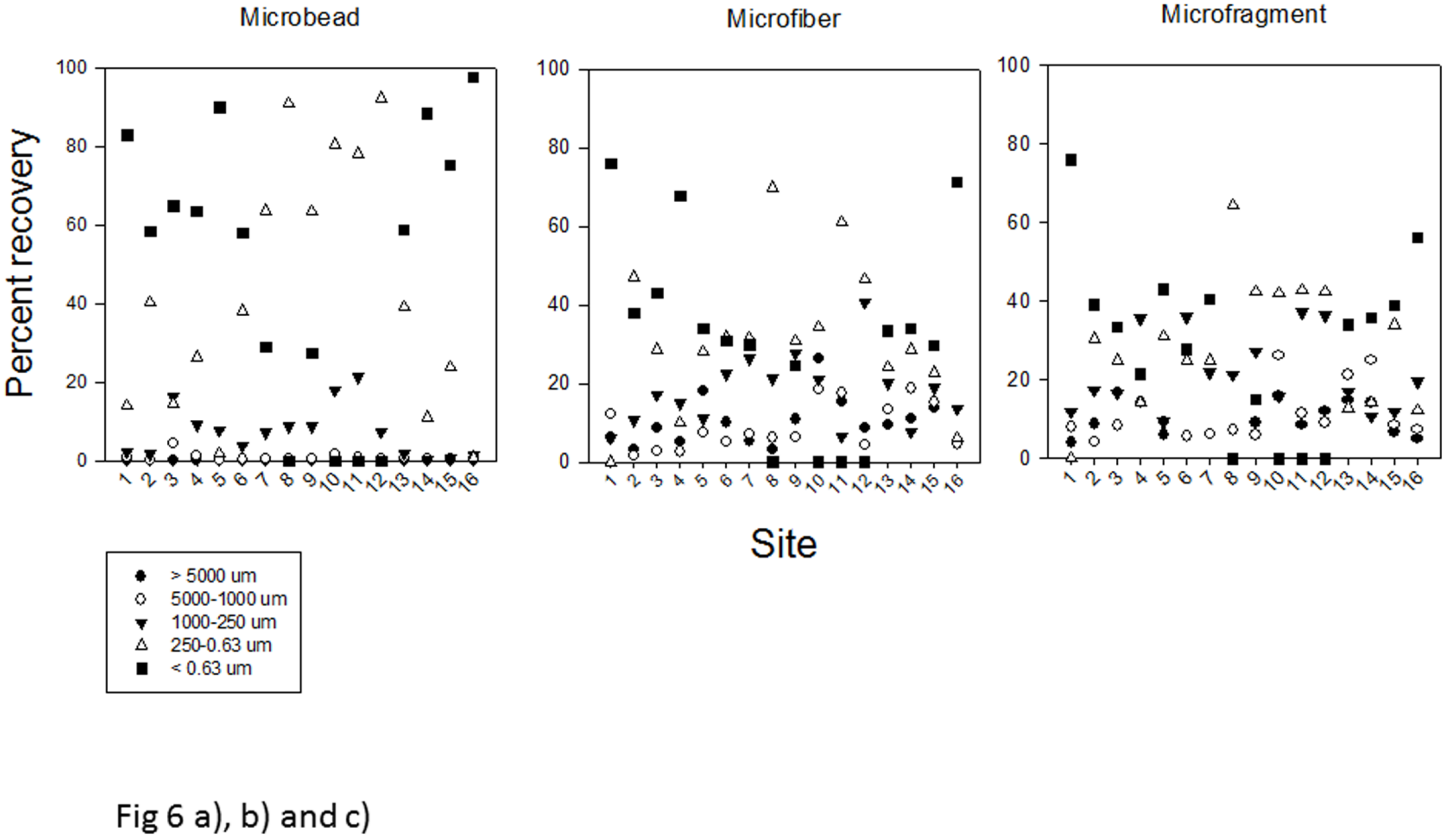
(a, b and c.) Percent abundance of microbeads a), microfibers b) and microfragments c) from the 5 sediment size fractions for the 16 sampling sites. Note how grain size grades of <0.63 μm and 250–0.63 μm contain the greatest number of microplastic particles.

Principal Component Analysis and analysis of similarity separated sites Royston Low Tide, Henry Bay Low and High Tide, Ferry Terminal Low and High Tide, and Metcalfe Bay Low Tide (sites 2, 5, 6, 13, 15, and 16, Fig 1b) from all other sites based on the number of total microbeads/kilogram sediment with the first principal component accounting for 84 percent of total variation among sample sites (Fig 5). Of note, at sites 1–6 and 13–16, those sites within Baynes Sound, 60 percent and greater of the microbeads were recovered in the 0.63 μm size fraction, whereas at sites 8, 10, 11 and 12 located within Lambert Channel, microbeads occurred in the next size fraction up, 250–0.63 μm (Fig 6a). Microfibers were generally represented in all size fractions with the exception of sites 4 and 16 where the majority occurred in the < 0.63 μm size fractions and sites 8 and 11 where greater than 60 percent occurred with the 250–0.63 μm size fraction (Fig 6b). As with microfibers, microfragments were recovered from all size fractions with the exception of site 1 and 16 where more than 50 percent were in the <0.63 μm and at site 8 where 65 percent were recovered from the 250–0.63 μm fraction (Fig 6c). Complete results of the PCA are presented in S2 Table.

Sites 8, 10, 11 and 12 occur on the west side of Denman Island within Lambert Channel, whereas sites 1–6 and 13–16 occur on the east side of Denman Island and the west side of Vancouver Island (Fig 1b) i.e., Baynes Sound. The discovery of two sizes of microbeads suggests two independent sources of microbeads to this region of coastal British Columbia. For Lambert Channel, microbeads would originate from sources within the Salish Sea, east of Denman Island. Baynes Sound however, has since the 1940’s been actively farmed for shellfish, notably the Pacific oyster with 50 percent of British Columbia’s product coming from this region. Previous research has demonstrated the presence of microfibers directly linked to industry practices [33], and it is likely that microbeads too originate from this source. Other possible sources of microbeads include expanded polystyrene (e.g. Styrofoam) used for flotation purposes [34], which can over time decompose into smaller particles, abrasives used for cleaning boat hulls and equipment [35], and personal care products [29]. Another potential source of microbeads to those sites where the greatest numbers were found (2 and 5), is the Courtney-Comox estuary (Fig 1b). Previous studies [24,36] on the depositional patterns of mud and gravel within Baynes Sound and the bathymetry of the region indicate that due to the presence of the “Comox Bar”, circulation patterns within this region of the sound would suggest that Royston and Henry Bay would be sites of greatest accumulation for materials possibly originating from the estuary. Indeed, Klein et al. [19] in their study of the occurrence and spatial distribution of microplastics in river shore sediments of the Rhine-Main Area in Germany underlined the importance of rivers as vectors of transport of microplastics into the ocean.

In addition to the shellfish aquaculture industry introducing microbeads into the intertidal environment, the industry also makes extensive use of High Density Polyethylene (HDPE), in the form of netting, oyster bags, trays, cages and fences (e.g., vexar) [37]. Each year, 3–4 tonnes of debris, comprised primarily of these plastic materials is recovered from the intertidal regions of Baynes Sound [38]. Sites where the greatest number of microfragments and microfibers were found (sites 5 and 15, and sites 13 and 15 respectively) also coincide with regions of extensive shellfish aquaculture equipment. Greater numbers of microfragments recovered from these regions could be a consequence of the continual mechanical breakdown of the HDPE materials over time and their subsequent accumulation within intertidal sediments. As well, recent research on the distribution of microplastics within the water column of Baynes Sound found concentrations of approximately 4000–5000 m^3^ of which 80% were fibrous [39]. Examples of shellfish materials that could contribute to this high microfiber load include ‘‘oyster blue” plastic rope, and ropes used for netting and longline culture.

Key determinants that control metal bioavailability to sediment ingesting organisms are grain size distribution and organic matter [40]. Sediment-associated contaminants tend to accumulate in depositional areas on small, fine-grained particles which have the highest surface area to volume ratio of any particle size class and tendency for higher concentration of organic matter [40]. Estimates of the percent mass of sediment microbeads indicated that amounts of microbeads present were as important on a weight percentage basis as compared to amounts of percent silt (< 0.63 μm) and percent organic matter at Royston Low Tide, Henry Bay Low and High Tide (sites 2 and 5/6) (Table 1).

**Table 1:** % organic matter (%OM), % silt, total number of microbeads recovered from sediment and % microbeads for minimum and maximum polymer densities for the 16 study sites. Two sites (values bolded, 2, Royston, Low Tide and 5/6, Henry Bay, Low and High Tide contained a percent mass of microbead equal to or greater than either %OM or % silt, two key sediment components which govern trace metal behavior within coastal sediments.

Site	% OM	% silt	Total number of micro-beads recovered from sediment	% Microbeads Density 1.34	% Microbeads Density 0.9
1	2.7	4	2712	0.5088	0.3417
**2**	**4.9**	**6.5**	**25368**	4.759	3.1964
3	2.2	2.8	540	0.1013	0.068
4	1.8	4.1	1989	0.3731	0.2506
**5**	**1.3**	**3.2**	**19806**	3.7156	2.4956
**6**	**1.3**	**1.7**	**9636**	1.8077	1.2141
7	1.1	2.3	4192	0.7864	0.5282
8	1	1.3	2531	0.4748	0.3189
9	1.2	1.4	4087	0.7667	0.515
10	2.1	0.4	945	0.1773	0.1191
11	2.2	0.5	1873	0.3514	0.236
12	1	0.8	1706	0.32	0.215
13	8.6	4	5798	1.0877	0.7305
14	7.1	10.2	8198	1.5379	1.0329
15	8.2	14.5	10463	1.9629	1.3183
16	5.3	9.2	10618	1.9919	1.3379

Microbeads are effective sorption surfaces for trace metals. Ashton et al. [41] determined the association of metals with plastic production pellets (PPP), sampled from four beaches in SW England and noted that pellets were enriched with cadmium and lead at two sites and that the PPPs accumulated metals to concentrations approaching those of sediment and algal fragments. Holmes et al. [42] assessed the interactions between trace metals and PPPs under estuarine conditions and concluded that plastic pellets effectively sorb trace metals and that PPPs may represent an important vehicle for the transport of metals in the marine environment. Rochman et al. [43] compared the long-term sorption of metals among plastic types in seawater and found that in general all types of plastics tended to accumulate similar concentrations of metals and that over a 12 month study period the concentrations of all metals increased over time and did not reach saturation. Thus at Royston and Henry Bay, microbeads may be the key determinant of trace metal behavior as compared to site highs in organic matter such as Metcalfe Bay.

Baynes Sound is home to a major shellfish industry of which 80 percent of the product is oysters and the remaining 20 percent scallops *(Patinopecten yessoensis*) and Manila clams (*Venerupis philippinarum*) [23]. Also commercially important is the varnish clam (*Nuttallia obscurata*) although it is not intentionally farmed, but rather is an invasive species that now dominates much of the intertidal clam community. All of these species filter and/or deposit feed on suspended materials which include algae and suspended sediments. Yang et al. [20] compared microplastics within 9 commercially important shellfish and noted that the type of microplastic recovered was species dependent. Fibers were the most common in blue mussels (*Mytilus spp*.) and Manila clams (63 percent of recovered particles from mussels and clams) whereas microbeads were found exclusively in oysters (60 percent of recovered particles from oysters). It is likely that oysters within this region of Baynes Sound are at risk of exposure via diet to microfibers and microbeads. Indeed, recent studies of Davidson and Dudas [44] confirmed the presence of microfibers in Manila clams sampled from 6 sites within Baynes Sound. These authors attempted to differentiate between clams sampled from 3 shellfish farms versus those from 3 “reference” sites in hopes of assessing if the shellfish industry was contributing to the burden of microplastics recovered from the clams. The widespread contamination of Baynes Sound however, precludes a “reference” site, and that both wild and farmed clams had ingested similar numbers of fibers given the wide spread contamination of this region is not surprising. Of real concern are the recent studies of Sussarellu et al. [22]. These authors found that oyster reproduction was adversely affected by exposure to polystyrene microplastics by interfering with energy uptake and allocation, reproduction and overall performance. Oysters exposed to microbeads have also been shown to be less fit with respect to reproduction and overall health [22].

## Conclusions

**Fig 7:**
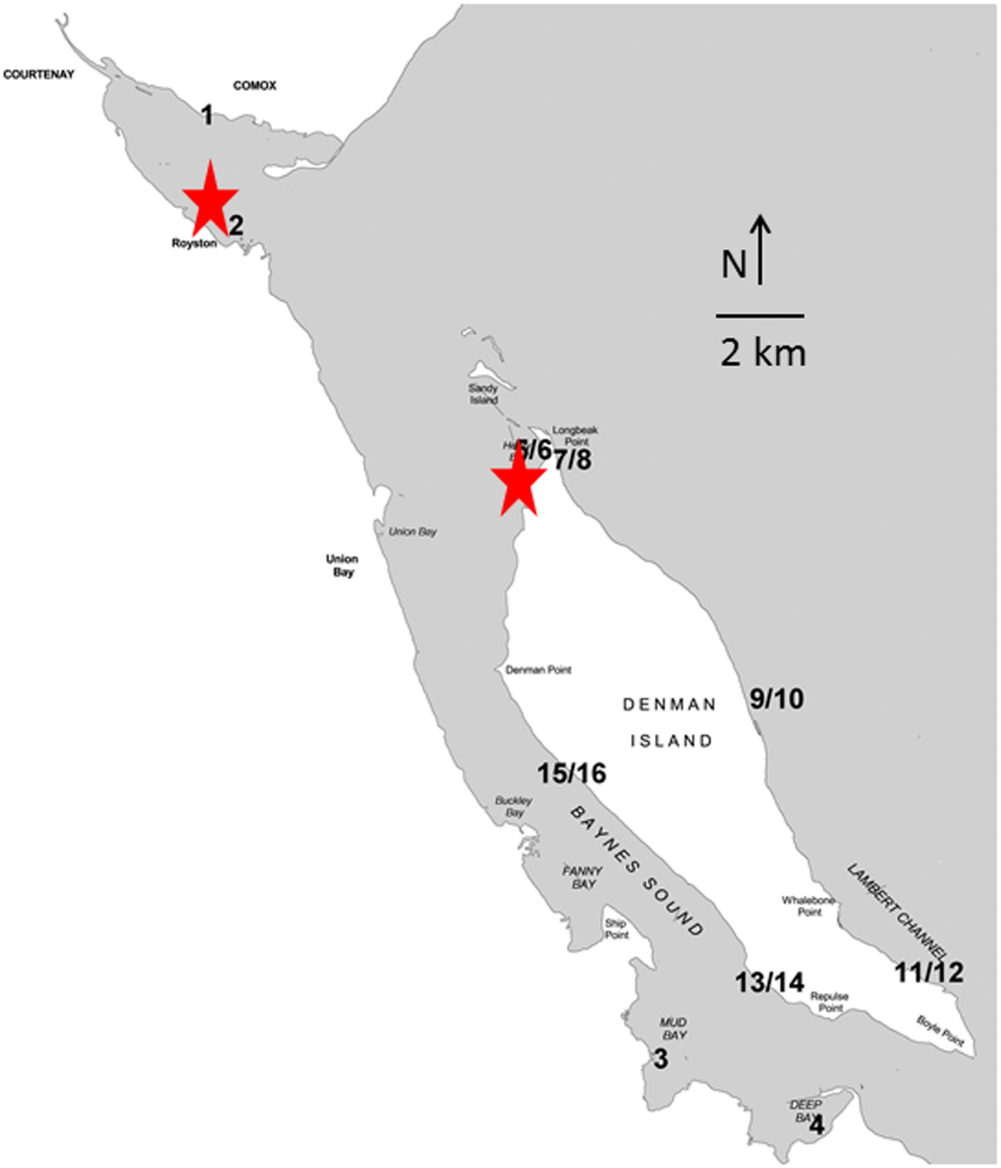
Sites of greatest microbead accumulation, likely a consequence of the Comox Bar a shallow underwater plateau that extends north of Denman Island which reduces circulation at the north end of Baynes Sound with this portion of the sound behaving like an embayed water body rather than an open channel [24].

We have shown that BC’s premier oyster growing region is highly contaminated with microplastics, notably microbeads at two locations within the sound (Fig 7). Sources of the microplastic include the shellfish industry as well as possible inputs from the Comox Estuary which receives urban input from the adjacent towns. It would be prudent to assess the degree to which oysters from this region are ingesting microplastics. If so, it would have direct implications for Canada’s oyster farming industry and sets an example for other shellfish growing regions of the world.

## Supporting information

S1 Table PCAforBS. (XLSX)

S2 Table Baynes Sound_15pl. (XLS)
